# The Characteristics and Distribution of Nerve Plexuses in the Dartos Fascia From Concealed Penis Children

**DOI:** 10.3389/fped.2021.705155

**Published:** 2021-08-05

**Authors:** WenFang Huang, DaXing Tang, WeiZhong Gu

**Affiliations:** ^1^Department of Anesthesiology, National Clinical Research Center for Child Health, The Children's Hospital, Zhejiang University School of Medicine, Hangzhou, China; ^2^Department of Urology, National Clinical Research Center for Child Health, The Children's Hospital, Zhejiang University School of Medicine, Hangzhou, China; ^3^Department of Pathology, National Clinical Research Center for Child Health, The Children's Hospital, Zhejiang University School of Medicine, Hangzhou, China

**Keywords:** nerve ending distribution, dartos fascia, concealed penis, penile shaft, urology

## Abstract

The purpose of this study is to analyze the nerve plexus distribution in dartos fascia of concealed penis (CP). A total of 28 CP patients met ASA categories I and II were included, with median age of 3.5 years (8 months−5 years). During the surgery, tissue samples of dartos fascia at points 3, 6, 9, and 12 o'clock of the penile shaft were collected. Standard hematoxylin and eosin (H&E) staining and S-100 immunohistochemical staining were used to analyze the nerve plexus distribution among different positions. The number of nerve plexuses in superficial fascia collected at the 6 o'clock position of the penile shaft was the most abundant among four positions (median 7.25, range 1–24). The abundant nerve plexuses in the dartos fascia of CP patients, especially at the 6 o'clock position, indicate that the surgery on the preputial frenulum should avoid damage to the dartos fascia, as it might be related to maintain the erection and sexual function in adolescence.

## Introduction

**Figure 1:**
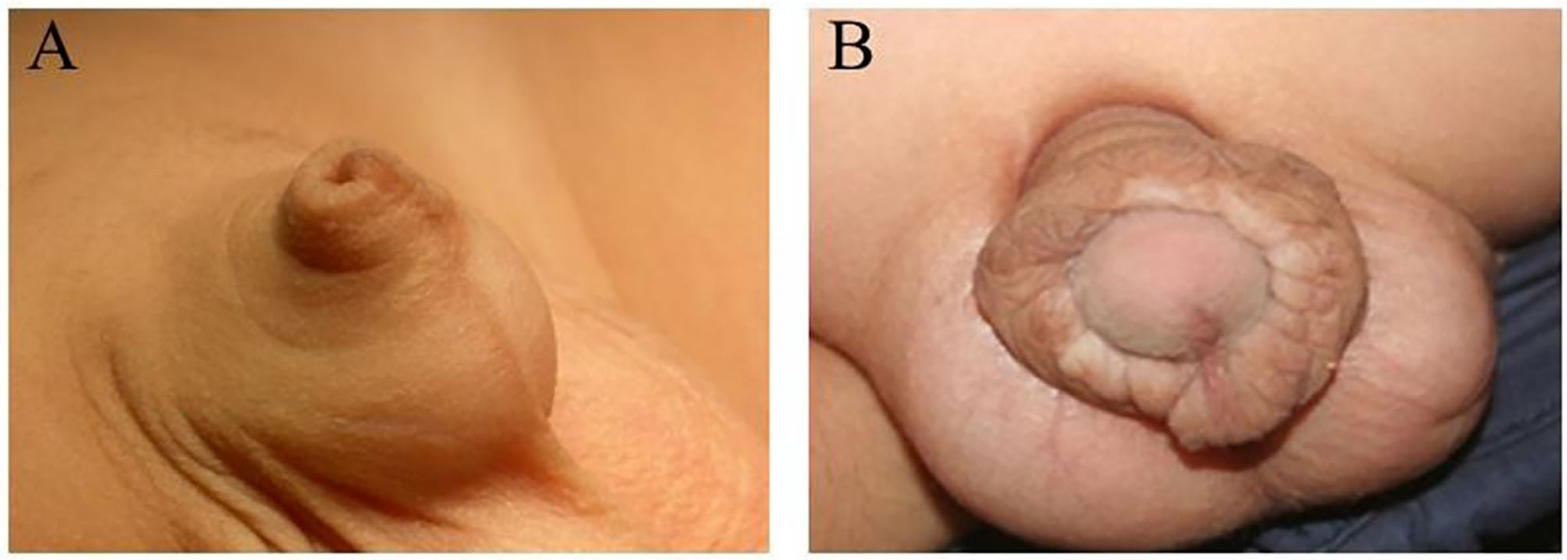
**(A)** Typical appearance of the concealed penis. **(B)** Proliferation of inner plate of the prepuce after the reconstruction of concealed penis.

Concealed penis (CP) is a relatively rare congenital malformation in children and has many unclear causes. The histological characteristics of nerve plexuses, especially dartos fascia in the CP, have not been well-explored. A normal-sized penis is surrounded by skin, subcutaneous tissue, and/or fat in the prepubic area (1). CP patients usually present with short and obvious phimosis (2–4) (Figure 1), but most patients have a normal penis length. Although a Japanese study reported that the prevalence in newborns was 3.7%, CP incidence has not been systematically studied (5). In addition to the abnormal appearance, CP may also have symptoms and adverse psychological effects on children and their parents, such as anxiety and/or depression (6). Spinoit et al. showed histological abnormalities of the dartos fascia in 74% of CP patients, and the dartos layer of the penis then became inelastic, thereby preventing the forward extension of the penis and trapping it under the pubic bone (7). Generally, CP surgical procedures include complete penile peeling, removal of excess suprapubic fat, reconstruction of penile skin with local flaps, and fixation of penile skin at the pubic bone and scrotal corner (2, 7, 8). These surgical techniques have reported satisfactory results with almost no complications (8–10). However, excessive defrosting of the foreskin may lead to a postoperative penile retraction, inversion of the foreskin caused by a lymphocytic obstruction (short-term), and long-term hypodermic bursa hyperplasia (long-term), which may lead to the poor appearance of the penis in some cases (9, 11, 12). Lim et al. revealed that the removal of abnormal dartos fascia completely removes the excess dartos tissue during the operation, which is a simple technique to avoid these complications (13). However, the elastic dartos fascia's surgical treatment is considered a universal surgical correction approach for CP patients (1).

CP remains a challenge for urologists, and flexible surgical approaches may be required (2). New surgical techniques and modifications are developing and being applied to the correction of this anomaly with good clinical outcomes (14–19). However, the nerve plexus distribution in dartos fascia in CP remains unclear. The histological examination of nerve plexus distribution of dartos fascia could reveal whether nerve damage will occur during dartos resection and may provide clinicians with appropriate surgical techniques to improve CP in children. Therefore, the histological structure and nerve plexus distribution in dartos fascia of the CP were studied. The results could provide valuable information for surgeons performing large-scale surgical removal of the fascia under the penis to maintain the appearance of the penis after the operation and reducing postoperative lymphatic drainage and scarring of the penis.

## Methods

### Ethics Statement

This prospective study was conducted on patients undergoing penile reconstruction surgery in the Children's Hospital, Zhejiang University School of Medicine, from January to July 2017. Dartos fascia samples were collected from these patients. This study was approved by the Ethics Board of the aforementioned hospital. The parents or legal guardians of the patients participating in this study also provided written informed consent. The samples were obtained from discarded tissues and have been mentioned in the informed consent.

### Subjects

Patients were diagnosed by experienced urologists. The inclusion criteria were ASA (the physical status scale of the American Society of Anesthesiologists) category I and II CP patients. Exclusion criteria were as follows: (1) patients with micro-penises (the penis length is <2 or more standard deviations below the mean), as this situation represents an endocrine problem; (2) patients with CP due to obesity; and (3) patients with atypical CP without penile reconstruction surgery.

### Surgical Procedure

After the patient was deeply anesthetized, a hemostat was applied at 0 and 12 o'clock positions at the penis for traction. A circumferential incision was made 0.5 cm proximal to the coronal sulcus. The penis shaft was completely degloved along Buck's fascia to the base, and the dysplastic dartos was resected. Then a V-shape incision at the ventral skin of penis was made. After the penis is pulled out from the incision, the white membrane and the dermis were sutured and fixed at the 12 o'clock position of base of the penis with 5-0 prolene suture, and the penis was pushed back. The midline between the ventral prepuce and frenulum was sutured. The urethral fascia and subcutaneous fascia at ventral penis were fixed with 5-0 suture to reconstruct the penis–scrotum triangle. Redundant tissues were excised. The inner and outer preputial skin and the incision of the ventral penis were interruptedly sutured with 6-0 absorbable sutures (Maxon). Compression dressing was applied.

### Histology

After the penis was detached, a 0.3 × 0.3 cm sample of dartos fascia samples was histologically examined for nerve changes using conventional hematoxylin and eosin (H&E) staining and S-100 immunostaining. The dartos fascia sample was fixed in 10% formalin solution for 12 h. After fixing, specimens were embedded in dehydrated paraffin and cut into 3–4-m-thick sections. H&E staining and S-100 immunoperoxidase staining were performed using S-100 antibody (rabbit polyclonal antibody with 1:1,000 dilution; Dako, Santa Clara, CA, USA). Microscope examination of H&E staining showed that the nucleus was blue and that the cytoplasm and extracellular matrix were pink in varying degrees. The nerve plexus was counted manually under the microscope.

### Statistical Analysis

The number of nerve plexuses was expressed as the median (range: min–max) at 3, 6, 9, and 12 o'clock positions. The Kolmogorov–Smirnov test was used to evaluate whether the data were normally distributed. The Wilcoxon rank test was used for the non-normally distributed variables, and the data were presented as the median. The non-parametric method Wilcoxon signed-rank test was used to compare different nerve plexus parts. All statistical evaluations are two-tailed, and *p* < 0.05 is considered significant. The statistical product and service solution (SPSS) statistical software version 22 was Windows (IBM Corp., New York, USA) for data analysis.

## Results

**Figure 2:**
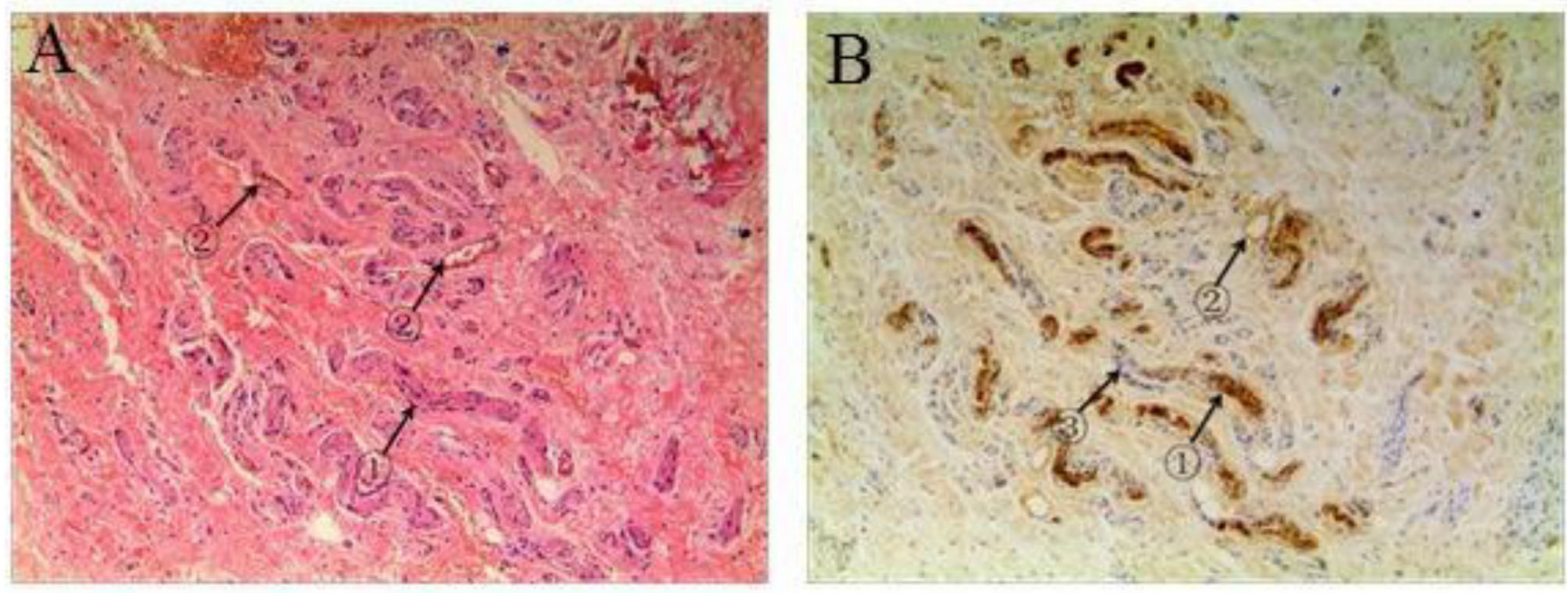
Staining for nerve plexus distribution in the superficial fascia dartos of the penis. **(A)** A representative hematoxylin and eosin (H&E) staining in a concealed penis (CP) patient showing ① nerve and ② blood vessels. **(B)** A representative S-100 positive straining of a CP patient showing ① nerve, ② adipocyte, and ③ nucleus of vascular smooth muscle. Images under ×50 visual field.

A total of 28 cases were included in this study. The median age of the CP patients was 3.5 years (range from 8 months to 5 years). No complaint about voiding function was reported after the surgery. H&E staining showed that nerve plexuses were distributed on the dartos fascia of the penis in the CP (Figure 2A). The results of S-100 immunostaining showed that there were many complete nerve plexuses in the penile dartos fascia in the CP (Figure 2B), of which the ventral region in the dartos tissue was the most concentrated. After the pairwise comparisons was conducted, no significant differences were found among the numbers of nerve plexuses at the 3, 9, and 12 o'clock positions; and the number of 6 o'clock position was significantly greater than that of 3, 9, and 12 o'clock positions (*p* < 0.05) (Table 1).

**Table 1:** Distribution of nerve plexuses (count) in various positions of the penis.

**Position (o'clock)**	**Median value**	**Minimum value**	**Maximum value**	p^*^-value
3	5.50	1.0	11.0	0.017^*^
6	7.25	1.0	24.0	–
9	5.00	2.0	15.0	0.020^*^
12	4.00	1.0	11.0	0.001^*^

* *p, the p-value obtained after comparison with the value at the 6 o'clock position*.

## Discussion

In this study, the samples collected at 6 o'clock showed abundant nerve plexus distribution than other positions. Anatomy and histology of the penis in a cross section showed that the nerves at 12 o'clock of the penis were absent, and the area around the frenulum of the most sensitive part of the penis was located at 6 o'clock (20). However, studies on the placement of reinforcement sutures at the quasi-cross angle have shown that placing three sutures at a 120° angle is sufficient to support the penis and position (21), which might cause interruption of penile sensation. Liu et al. reported a surgical technique for attaching the dartos facial band to the distal or central shaft of the penis, which may be a better surgical approach to preserve the dartos plexus (22). Whether removing the peripheral nerves, especially the frenulum, can lead to postoperative complications, such as sexual dysfunction still needs to be further explored.

According to penis anatomy, the innervation of the penis is composed of several branches of the pudendal nerve derived from S2-4, including the dorsal, cavernous, and perineal nerves of the penis (23). After branching from the pudendal nerve, the dorsal nerve penetrates the back of the penis and Buck's fascia. The dorsal nerve is located above the cavernous body, combining with the dorsal arteries and veins and supplying the penis skin, including the glans (24). The nerve fibers of the dorsal penis pass through the cavernous body of the penis, innervate the urethral cavity as an afferent nerve, receive input, and transmit information during ejaculation or urination. The perineal nerve also branches out of the pudendal nerve, innervating the dry ventral skin, frenulum, and cavernous body. The cavernous nerve branches from the autonomic pelvic plexus are accompanied by the prostate neurovascular plexus and carried the sympathetic and parasympathetic nerve fibers to the cavernous body (25). These nerves transmit sensory signals to the central nervous system, which is the key to achieve an erection and sexual function (26).

The general principle of CP surgery is to remove the sheath along Buck's fascia altogether and correct the defects of the dry skin of the penis (21, 22) to restore normal erection and sexual function. Different surgical results reflect different understandings of the cause, and many techniques have been described to correct CP. In children, CP is usually caused by the inelasticity of the infants' dartos fascia (27). Looking back and comparing with the current literature, it has been reported that multiple dartos and Buck's fascia dissections and chord dissections have been performed in children with cervical lymph nodes (21). Some studies also believe that the key to correcting CP in children is to release abnormal dartos and fix the skin of the penis on Buck's fascia (13, 28). In this study, abundant nerve plexuses were observed on children's dartos with CP. It was also reported that removing the dartos to correct CP may result in loss of sexual sensation due to removing a large number of nerve plexuses (29). Besides, the concentration of substance P and calcitonin gene-related peptide in nerve terminals related to penile afferent sensation was higher in the frenulum compared with other parts (30). Although removing the superficial fascia has been shown to improve the children's penis appearance, it may reduce the sensitivity of the penile skin after puberty.

Circumcision is traditionally used in CP surgery, but the problem of insufficient skin coverage is frequently seen after the surgery, especially in cases having undergone circumcision as the initial treatment option (31–34). Besides, following circumcision, the penis recedes further beneath the surrounding tissues, giving the impression that an inadequate circumcision was performed, and so, many of these children are then referred for redo circumcision (31–33, 35–37). In this process, many surgeons choose to remove many dartos attached to Buck's fascia to reduce the postoperative edema or hyperplasia of the dartos, resulting in poor cosmetic results. This research aims to determine whether this manipulation is harmless. If not, the modification of the operation might be suggested to save the dartos. Besides, a penile surgery combined with an additional caudal block on the ventral side of the penis in this study can effectively relieve the pain. Whether the satisfactory results could also apply to caudal block for hypospadias repair in children still needs to be explored.

This study was limited by the small sample size, and CP in different ages was not included, such as during adolescence. In addition, long-term research on erectile function and sexual function will be needed to get further conclusion.

## Conclusion

In this study, surgical procedures of CP children were modified to keep almost all dartos under the foreskin because abundant nerve plexuses were observed in the superficial fascia. Therefore, it is suggested that the surgical treatment of CP children should not only consider cosmetics but also avoid the possible damage of the superficial fascia dartos, especially at the frenulum, to maintain the erection and sexual function of the penis in adolescence.

## Data Availability Statement

The original contributions presented in the study are included in the article/supplementary material, further inquiries can be directed to the corresponding author.

## Ethics Statement

This study was approved by the Ethics Board of the above hospital. Written informed consent to participate in this study was provided by the participants' legal guardian/next of kin.

## Author Contributions

WH: guarantor of integrity of the entire study, study concepts, study design, definition of intellectual content, literature research, clinical studies, experimental studies, data acquisition, data analysis, statistical analysis, manuscript preparation, and manuscript editing. DT: guarantor of integrity of the entire study, study concepts, study design, definition of intellectual content, literature research, clinical studies, experimental studies, data analysis, data analysis, statistical analysis, manuscript preparation, manuscript editing, and manuscript review. WG: clinical studies, experimental studies, and data acquisition. All authors contributed to the article and approved the submitted version.

## Conflict of Interest

The authors declare that the research was conducted in the absence of any commercial or financial relationships that could be construed as a potential conflict of interest.

## Publisher's Note

All claims expressed in this article are solely those of the authors and do not necessarily represent those of their affiliated organizations, or those of the publisher, the editors and the reviewers. Any product that may be evaluated in this article, or claim that may be made by its manufacturer, is not guaranteed or endorsed by the publisher.
